# In Vitro Techniques to Domesticate Mortiño (*Vaccinium floribundum* Kunth) and Other *Vaccinium* Species: A Review

**DOI:** 10.3390/plants14111596

**Published:** 2025-05-24

**Authors:** Nataly Tatiana Coronel Montesdeoca, Guillermo Alexander Jácome Sarchi, Rafael Martínez, Francisca Hernández

**Affiliations:** 1 Grupo de Investigación Agricultura Sostenible (GIAS), Carrera de Agropecuaria, Universidad Politécnica Estatal del Carchi, Tulcán 040102, Ecuador; nataly.coronel@upec.edu.ec (N.T.C.M.); guillermo.jacome@upec.edu.ec (G.A.J.S.); 2 Grupo de Investigación en Fruticultura y Técnicas de Producción, Instituto de Investigación e Innovación Agroalimentaria y Agroambiental (CIAGRO-UMH), Universidad Miguel Hernández, Carretera de Beniel, km 3.2, 03312 Orihuela, Alicante, Spain; rafa.font@umh.es

**Keywords:** micropropagation, callogenesis, axillary buds, seed germination, culture media

## Abstract

*Vaccinium floribundum* Kunth, also known as mortiño, is of cultural, gastronomic, pharmaceutical and ecological importance in the Andes due to its regenerative capacity to preserve vegetation after destructive fires. The main limitation for the production of mortiño fruits is that the plant has not been domesticated or cultivated, which could pose risks to the species and the paramos where it lives. In vitro culture is a crucial technique for propagating horticultural crops where factors such as the concentration, growth regulators, medium and explant parameters must be optimized to ensure the success of in vitro propagation techniques. This review uses the Prisma methodology, identifying 47 studies on the in vitro cultivation of *Vaccinium*, but only five studies on the domestication of *V. floribundum* Kunth using three in vitro cultivation techniques (axillary buds, seed germination and induced callogenesis) were published in Scopus and ScienceDirect. Therefore, the objective is to provide information on in vitro propagation techniques for the domestication of *V. floribundum* Kunth.

## 1. Introduction

In recent years, biotechnology has become one of the most talented scientific disciplines, which is why plant biotechnology is a constantly evolving area of study [1]. Biotechnological methods, including plant cell and tissue cultures and the genetic modification of plants, are increasingly used in the in vitro laboratory to produce high-quality PDMCs (plant-derived medicinal compounds) [2].

In vitro culture has superior commercial value in the accelerated production of clonal plants compared to traditional propagation methods. In addition, it has proven to be of great practical value in the development and conservation of disease-free planting materials, germplasm preservation, and as a complement to conventional methods of plant advancements, such as plant genetic engineering. The discovery and use of modern tissue culture techniques are considered to be paving the way for a second ecological revolution [3].

Lately, in vitro propagation technology of vegetative and recalcitrant plants has become a powerful industry to meet the needs of the agricultural sector [1]. Micropropagation is a crucial procedure for the mass propagation of clones and a tool for in vitro research. An initial step to go through in this procedure is the implementation of new explants in vitro [4]. The clonal propagation of plants is carried out through tissue, cell and organ culture techniques [5].

The means of maintaining or growing tissues in vitro to promote the differentiation and preservation of structure and/or function is called tissue culture [5]. Plant tissue culture (PTC) is a technique that has great potential as a means of vegetative propagation of economically important species [1]; i.e., it refers to the aseptic culture of tissue and organ explants in closed containers using established culture methods in a controlled environment [5]. In addition, it is a model system for investigating physiological, biochemical, genetic and structural problems related to plants [1].

Gottlieb Haberlandt was recognized as the forerunner of plant cell and tissue culture in 1902 as he founded the theory of totipotency in the plant cell. Although his first attempt to culture hair cells from the mesophyll tissue of the monocotyledonous plant leaf failed due to the absence of growth regulators necessary for cell division, he did not succeed in this attempt [6].

The advantages and applications of PTC as a technique are considered to be in constant growth, as it can be cultivated in the absence of bacteria, fungi and viruses; in addition to preserving substances, its germplasm is used as a tool for genetic improvement [1]. Plant tissue culture provides an alternative for rapid large-scale propagation, producing pathogen-free plants, increasing the synthesis of useful metabolites and the conservation of germplasm [5].

This is why the application of in vitro cultures overcomes the restrictions linked to the conventional propagation of seedlings [5] and provides an option to boost plant growth throughout the year, thereby increasing production efficiency and profitability [7].

In addition, it has become a reliable method for the mass production of plant materials, as the market for innovative plant-derived methods of medicinal use has grown significantly. Also, since it allows for the manipulation of biosynthetic pathways to drive the production and accumulation of certain compounds, this technology has enormous potential for the manufacture of natural bioactive chemicals [8].

There are two streams in the progress of in vitro plant tissue technologies: in the scientific field, where they constitute a model for fundamental research, and in the commercial field, where they are routinely used in an increasing number of plant species. Micropropagation is the most widely employed technique, and its uses are oriented towards plants that are propagated vegetatively. Depending on the species and growing circumstances, in vitro propagation can be achieved through the following procedures: (1) axillary shoot proliferation, (2) node culture, and (3) the creation of new adventitious shoots through shoot organogenesis and somatic embryogenesis [5,6].

The implementation of in vitro plant biotechnology in berry crops is based on the existence of effective regeneration protocols that are adjusted to the genotype and on the correct mix of exogenous hormones (auxin and cytokinin) incorporated into the medium [9]. In vitro blueberry tissue culture facilitates rapid replication through internodal propagation and is also essential for all efforts that require sterile starting materials, such as gene transformation and, more recently, gene editing [2].

The application of biotechnology, especially genetic modification, is less common in conventional breeding of *Vaccinium* varieties [10]. However, efficient genetic transformation systems are crucial for mechanistic studies of poorly known genes and for improving traits such as yield, quality, and resistance to biotic/abiotic stresses [10]. Genetic transformation is essential to confer desirable traits not present in the native *Vaccinium* genome [10]. The *Agrobacterium tumefaciens*-mediated transformation method has been successfully applied in *Vaccinium* varieties using leaf disks [10]. This method has also been reported in *Fragaria* spp. [9]. Reporter genes such as GUS (β-glucuronidase) and GFP (green fluorescent protein) as well as genes of agronomic interest such as Bar (herbicide resistance) and CBF (cold stress resistance) have been transferred. Transformation efficiency can vary significantly among genotypes [10].

There are numerous studies in the literature on the in vitro propagation of blueberries. However, all of the aforementioned efforts presented considerable fluctuations in terms of basal media, in addition to plant growth hormones, growth conditions, explant types, sampling and physiological conditions [11]. However, the most optimal plant growth can be achieved under in vitro conditions by using the appropriate medium composition and pH for the species [7].

According to the International Blueberry Organization, between 2016 and 2020, the global blueberry growing area increased from 132.56 to 205.67 thousand hectares. This represents an increase of 73.1 thousand hectares, mainly because of the growth of growing areas in China, Peru, Poland and other areas. In 2020, global blueberry production exceeded 850 thousand tons [12]. In 2023, the global blueberry cultivated area reached 267,000 hectares.

Wild berries are among the most important berry species in the world. They are a rich source of a wide variety of bioactive substances. They are in demand by the food processing and pharmaceutical industries because of their delicious taste and high bioactivity value [6]. Blueberries (*Vaccinium* spp.) are members of the family Ericaceae and belong to small fruiting crops [13]; their production has attracted increasing interest worldwide following the realization of their importance for diets and human health [14] including ≈400 other species [15].

*Vaccinium floribundum* Kunth is particularly common in the Andes from Venezuela to Bolivia [16] and is a woody plant that loses its leaves in response to dry seasons or seasonal changes; i.e., it is a semi-evergreen shrub [17]. The shrub can reach a height of 1.5 m, with 2 cm long lanceolate leaves and serrated edges that produce round, bluish-black berries about 8 mm in diameter [18]. This species inhabits climates with open mountain slopes. The maximum production of mortiño occurs between October and November, although in Ecuador, the fruit can be found in smaller quantities throughout the year [19].

*V. floribundum* Kunth, also called the Andean berry [18], is known in Ecuador as mortiño [17], grapes from the Andes, manzanilla del cerro, raspadura quemada and uva de monte [20]; in Perú as alabilí [21], macha macha [22], pushgay, uvitas and congama; and in Colombia also as mortiño, chivaco, agraz and agracejo [20].

In recent years, mortiño has been investigated for its importance as a gastronomic and cultural nutraceutical fruit, as well as for its high antioxidant capacity derived from its wide range of polyphenolic compounds [18]. On the other hand, it plays an important ecological role in the High Andes due to its regenerative capacity to preserve moorland vegetation after destructive fires caused by man [22]. In other words, it is a species of commercial, industrial, cultural and ecological interest that is in danger of extinction due to the continuous fragmentation of its natural habitat by anthropogenic processes such as deforestation, productive reconversion of land and overexploitation [16]. Therefore, the objective is to gather information on in vitro culture techniques to domesticate mortiño using efficient in vitro propagation methods.

## 2. Literature Review

**Figure 1:**
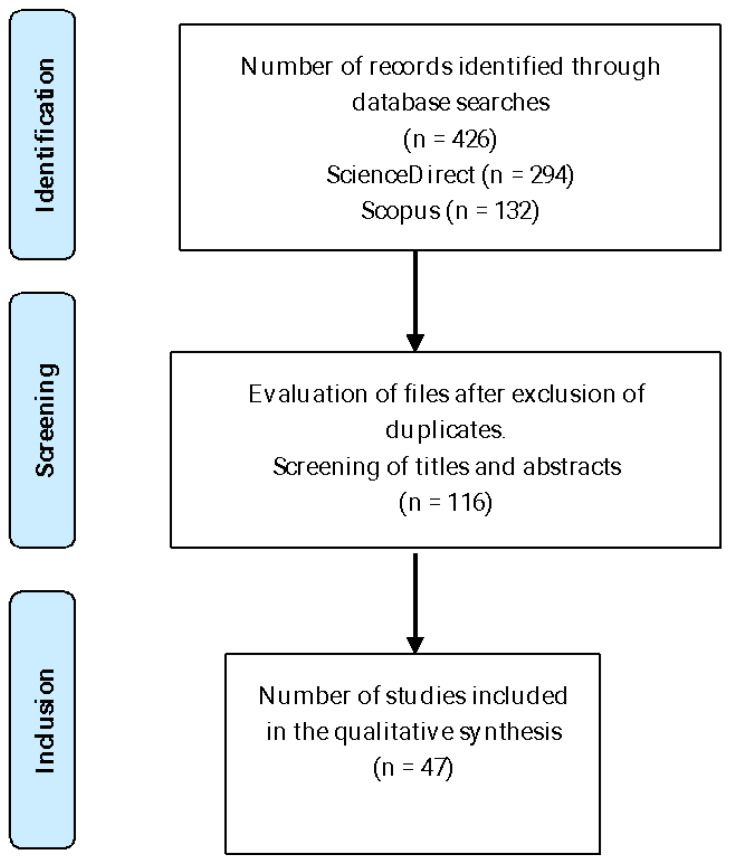
Diagram of selection of scientific publications.

The PRISMA 2020 statement was used in this review [23] to generate a comprehensive literature search in the ScienceDirect and Scopus databases up to January 2025 (Figure 1). The search was limited to scientific publications in English from 2014 to 2024 and focused on the following keywords: (“in vitro culture” OR “micropropagation” OR “tissue culture” OR “plant tissue culture”) AND (“*Vaccinium floribundum* Kunth” OR “*Vaccinium floribundum*” OR “blueberry” OR “*Vaccinium*” OR “fruit crops”).

Exclusion was performed using the criteria of (i) plant species studied, (ii) a study of chemical characteristics, (iii) medical applications and (iv) genetic studies. The systematic review addresses blueberry propagation, culture media and in vitro culture techniques to propagate *V. floribundum* Kunth.

In the present study, 426 scientific articles in English were identified through database searches, with 294 of them from ScienceDirect and 132 from Scopus. After a rigorous selection process, forty-seven studies were included in the qualitative synthesis. These selected studies are of vital importance, as they provide an in-depth and detailed view of the research topic, allowing for a more complete and nuanced understanding.

The inclusion of these studies not only ensures the quality and relevance of the data analyzed but also ensures that the conclusions derived are robust and based on solid evidence. Thus, of the forty-seven articles in the qualitative synthesis, only five studies deal with propagation techniques of *V. floribundum* Kunth, while the other forty-two articles deal with in vitro culture techniques of different blueberry varieties, which represent a fundamental pillar for the advancement of knowledge in this specific field.

## 3. Study Characteristics

This review identified five studies [16,17,19,24,25] that address the in vitro propagation of mortiño published up to December 2024. The studies analyzed were conducted in Ecuador where three articles [17,24,25] with in vitro propagation protocols of mortiño, one article [16] of the reproductive phenological stages of *V. floribundum* Kunth and one review article [19] have been published. We found (n = 42) articles using in vitro propagation for different blueberry varieties. The oldest study for blueberries was published in 2018 [17], and the remaining articles were published between 2022 and 2023. The selected studies included data on the reproductive phenological stages of *V. floribundum* [16], an efficient propagation methodology for *V. floribundum* by axillary bud growth [17], callogenesis as an alternative for the in vitro regeneration of mortiño plants [24] and an efficient in vitro protocol for accelerated seed production [25]. The review article dealt with the horticultural and biochemical aspects of mortiño [19].

The (n = 42) articles deal extensively with the micropropagation of various *Vaccinium* species and their hybrids, detailing in vitro protocols for multiplication, regeneration and rooting where various factors influencing the culture were investigated, such as media, growth regulators (cytokinins and auxins), pH, light and substrates. In addition, topics such as the induction of polyploidy by colchicine, the evaluation of the genetic fidelity of micropropagated plants and the use of bioreactors for large-scale production are addressed. Aspects such as ex vitro acclimatization and the application of agricultural residues in culture media are also considered.

### 3.1. Species Domestication

In the scientific literature reviewed (n = 47), several species of the genus *Vaccinium*, including the highbush blueberry (*V. corymbosum* L.), the rabbiteye blueberry (*V. ashei* Reade), the European blueberry (*V. myrtillus* L.), the blueberry (*V. floribundum* Kunth), the lowbush blueberry (*V. angustifolium* Ait.), *V. arboreum* and *V. uliginosum* L., have been the subject of studies in the context of in vitro culture.

The highbush blueberry (*V. corymbosum* L.) is an important species from a horticultural and nutraceutical point of view, with a growing demand in the market [26,27]. Numerous varieties have been developed, such as ‘Duke’, ‘Liberty’, ‘Meader’, ‘Blueray’, ‘Patriot’, ‘Farthing’, ‘Legacy’, ‘Elizabeth’ and ‘Hortblue Petite’, which differ in their response to in vitro culture [7,16,28,29]. This species is characterized by its need for soils with low pH, generally in the range of 3.0 to 6.0, with the optimum pH being around 5.0 for the proliferation of shoots [4].

Micropropagation studies aim to optimize the production of high-quality, virus-free shoots to meet the needs of growers, as well as to provide the material for gene editing and genetic transformation. Different types of explants, such as shoot apices, nodal segments of in vitro grown plants, leaves and micro-stems, have been used for the initiation of in vitro cultures [7,8,27,28,30]. The response to culture is influenced by factors such as the type of culture medium (Woody Plant Medium—WPM and Chee and Pool—C2D), the concentration of growth regulators (BAP, 2iP and zeatin), the presence of structuring agents in the medium (agar, rice husk, coconut fiber and S-10 Beifort^®^) and the environmental conditions of incubation (photoperiod and temperature) [7,8,11,27,29].

The rabbiteye blueberry (*V. ashei* Reade) is another commercially important species. In vitro culture studies have focused on micro shoot proliferation and rooting ex vitro using different light spectra to optimize the process [31,32].

The European bilberry (*V. myrtillus* L.) is a species that grows wild in regions such as Austria and Turkey [33,34]. Research has compared in vitro establishment from mature and juvenile plant materials, showing significant differences in microshoot induction and rooting. Modified culture media, such as WPM, supplemented with various combinations of growth regulators such as IAA, GA3 and zeatin have been used [34].

Mortiño (*V. floribundum* Kunth) is a native species of the Andean highlands of South America, with potential for agroindustry due to its nutraceutical fruits. Conventional propagation strategies have proven difficult, which has prompted the development of in vitro culture protocols from seeds and axillary buds. Studies have evaluated different culture media (WPM and MS), photoperiods, temperatures and growth regulators (TZR, ZEA, 2iP, IBA, NAA and IAA) to optimize in vitro germination, multiplication and rooting, as well as ex vitro acclimatization [17,24,25].

The lowbush blueberry (*V. angustifolium* Ait.) has also been micropropagated using bioreactor systems, such as the RITA (Recipient for Automated Immersion Technique), using specific culture media for shoot proliferation [6]. *V. arboreum*, also known as the sparkleberry, has been investigated as a potential rootstock in the blueberry industry. A reliable micropropagation protocol has been developed for its rapid multiplication from nodal segments of axillary buds [35]. *V. uliginosum* L. (bog blueberry) is another species for which we have sought to establish efficient in vitro production protocols by using nodal segments as explants and evaluating different basal media (WPM, AN and MS) and growth regulators (zeatin, 2iP, TDZ, IBA and IAA) [36].

Finally, *V. arctostaphylos* L., an endangered medicinal plant, has also been investigated with the aim of developing a reliable propagation system for its conservation and commercial cultivation through nodal segments [37].

In the literature review (n = 5), the main current limitation for fruit production of *V. floribundum* Kunth is that the plant has not been domesticated or cultivated [17]. Failed attempts at domestication could pose risks, both for the species and for the moorlands where it lives [19]. As a result, human consumption of mortiño in Ecuador has decreased, as well as its availability in local markets [16]. Mortiño presents considerable limitations for its exploitation as a commercial resource and the conservation of its germplasm due to the difficulties in cultivating this species from seeds or propagating it using rooted cuttings. In addition, it is limited by its altitudinal and climatic requirements, which explains the need to implement micropropagation techniques [17]. To domesticate the mulberry tree, it is crucial to know the conditions in which this fruit species grows, such as its particular ecology, climatic requirements, soils and rhizosphere [16]. The establishment of an efficient propagation system would contribute considerably to the preservation of this valuable biological resource. Table 1 shows in which geographical locations the different species of *Vaccinium* spp. have been studied.

*Vaccinium* spp. have a wide geographical distribution, ranging from cold temperate regions, such as *V. corymbosum* in Poland and the USA, to high mountain ecosystems, such as *V. floribundum* in the Andean highlands of Ecuador or *V. uliginosum* in the alpine zones of Turkey. Therefore, the in vitro propagation of *Vaccinium* spp. must consider not only the intrinsic physiological conditions of each species but also their geographical origin and ecological adaptations.

### 3.2. Culture Media

Culture media play a crucial role in the in vitro propagation of blueberries, providing the nutrients and growth regulators necessary for explant development. Table 2 presents the most commonly used culture media with the main species of *Vaccinium* spp.

In the literature review, the culture media used in Vaccinium in in vitro micropropagation research are mainly composed of the Woody Plant Medium (WPM) (n = 47), Murashige and Skoog (MS) medium (n = 19), Anderson medium (AN) (n = 17), Chee and Pool medium (C2D) (n = 4) and BM-D culture medium (n = 4). WPM is the culture medium for woody plants originally formulated by Lloyd and McCown [39]. Since then, it has been used for the propagation of woody species thanks to its nutrient mixture composed of inorganic salts, vitamins, amino acids and carbohydrates [39].

The Woody Plant Medium (WPM) is frequently mentioned as one of the most suitable for the micropropagation of various *Vaccinium* species, including the highbush blueberry (*V. corymbosum* L.) [40], the European blueberry (*V. myrtillus* L.) [33], the bog blueberry (*V. uliginosum* L.) [36] and *V. arctostaphylos* L. [37]. It has been used as a basal medium for sprout initiation [33,36], the multiplication of shoots [33,36,37] and rooting [11,33,36].

WPM has proven to be more suitable for the micropropagation of highbush blueberry cultivars compared to the Anderson medium [13]. However, in some studies, shoot growth on WPM was lower than that obtained on DM or MW (a mixture of equal parts of DM and WPM) [13]. Modifications of WPM, such as mWPM (modified Woody Plant Medium), have been evaluated for the regeneration of *V. floribundum* [17] and for the in vitro establishment of *V. corymbosum* [4]. Variations in WPM nitrogen salts have also been tested [4].

WPM has been supplemented with different concentrations and combinations of growth regulators to optimize different stages of micropropagation. For example, it has been used with zeatin and IBA for shoot initiation [33,36], with different concentrations of zeatin, 2iP and TDZ together with IBA for shoot multiplication [33,36] and with different concentrations of IBA with or without activated carbon (AC) for rooting [33,36]. A concentration of 20 g L^−1^ sucrose in WPM under temporary immersion systems (TISs) has been found to be highly recommended for blueberry micropropagation, improving plant quality for acclimatization [31].

The Murashige and Skoog (MS) medium has also been used in *Vaccinium* spp. micropropagation [13,25,33,36,41]. WPM and Anderson have been compared for shoot initiation in *V. uliginosum* and *V. myrtillus*, with better results obtained with WPM in combination with zeatin and IBA [33,36]. It has been reported that shoot proliferation in the MS medium is slow, and shoots tend to show hyperhydricity [13]. In the context of in vitro seed germination of *V. floribundum*, MS was evaluated together with WPM, with WPM being the best treatment [25]. MW (a mixture of MS and WPM) has been used in studies of highbush blueberry shoot proliferation, obtaining in some cases better growth than WPM alone [11,13].

The Anderson Rhododendron (AN) medium [11,13,33,36,41] has been compared to WPM and MS for shoot initiation in *V. uliginosum* and *V. myrtillus*, showing lower shoot initiation compared to WPM with zeatin and IBA [33,36]. It has been used for in vitro rooting of highbush blueberry shoots supplemented with zeatin and IBA or IAA [11]. In multiplication studies of *V. arctostaphylos*, AN was compared with MS and WPM, with WPM being the most efficient medium [37].

The Chee and Pool (C2D) medium is mentioned in a study evaluating the effect of explant type, medium (C2D or WPM) and BAP concentration on ‘Legacy’ highbush blueberry shoot regeneration [27]. The BM-D medium was used in a study on adventitious shoot regeneration in *V. vitis-idaea* ssp. *minus* (lingonberry) on a semisolid medium supplemented with zeatin and TDZ [42].

The supplementation of these basal media with growth regulators such as auxins (e.g., IBA, IAA and NAA) and cytokinins (e.g., zeatin, 2iP, BAP and TDZ) is essential to control the different stages of in vitro culture, including initiation, multiplication and rooting [13,37,41]. In addition, the addition of other components such as activated charcoal to the rooting medium can promote root formation in some species [33,36,37]. The use of alternative structuring agents to agar, such as rice husk, coconut fiber and S-10 Beifort^®^, in the growing medium for highbush blueberry propagation has also been investigated [29].

**Table 2:** Efficient culture media used for the in vitro propagation of *Vaccinium* spp.

Vaccinium Species	Optimal Medium	Growth Regulators (Effective Dose)	Type of Explant	References
*V. uliginosum* × (*V. corymbosum* × *V. angustifolium*)	AN	1.0 mg L^−1^ 2iP for morphogenesis (sprout formation).	Seeds	[43]
*V. uliginosum* × (*V. corymbosum* × *V. angustifolium*)	1/2 AN	Hormone-free effective for rooting.	Microbrotes	[43]
*V. corymbosum* (High Blueberry)	WPM	0.5 mg L^−1^ zeatin (ZEA) for propagation and maintenance.	Segments of microshoots (1.0–2.0 cm)	[30]
*V. corymbosum* ‘Duke’	WPM con S-10 Beifort^®^	The 0 mg L^−1^ 2iP dose showed the highest multiplication rate.	Outbreaks	[29]
*V. corymbosum*	C2D o WPM	2.0–8.0 mg L^−1^ BAP for shoot development, varying according to the type of explant.	Shoot tips or two-node shoots.	[27]
*V. corymbosum* ‘Elizabeth’, ‘Liberty’ y ‘Meader’	AN o WPM	0.1 mg L^−1^ zeatin and 0.5 or 1.0 mg L^−1^ IBA or IAA for rooting.	Axillary shoots (2.5 cm)	[11]
*V. ashei* ‘Delite’ (Bilberry Rabbit’s Eye)	WPM	The 0.55 mg L^−1^ zeatin (ZEA) dose showed a high survival rate and good shoot formation.	Microbrotes	[4]
*V. arctostaphylos*	WPM	0.1 mg L^−1^ IBA and 2 mg L^−1^ Zeatin for high multiplication rate, shoot length and dry weight.	Nodal segments	[37]
*V. uliginosum* (Swamp Blueberry)	WPM	1.0 mg L^−1^ zeatin and 0.1 mg L^−1^ IBA for improved shoot initiation and elongation.	Nodal segments	[36]
*V. uliginosum*	WPM	2.0 mg L^−1^ zeatin, 0.1 mg L^−1^ IBA and 0.2 mg L^−1^ GA3 for the highest shoot length and number of shoots.	Lateral buds with one or two leaves	[36]
*V. uliginosum*	WPM	0.5 mg L^−1^ IBA and 1.0 mg L^−1^ activated carbon (AC) for the highest rooting percentage.	Microbrotes	[36]
*V. myrtillus* (Wild Blueberry)	WPM	1.0 mg L^−1^ zeatin and 0.1 mg L^−1^ IBA for sprout initiation.	Lateral buds	[33]
*V. myrtillus*	WPM	2.0 mg L^−1^ zeatin for multiplication.	Lateral buds	[33]
*V. myrtillus*	WPM	0.5 mg L^−1^ IBA and 1.0 mg L^−1^ AC for rooting.	Microbrotes	[33]
*V. floribundum* (Mortiño)	WPM	No regulators specified; optimal conditions: 24 h of light and 18 °C.	Seeds	[25]
*V. floribundum*	WPM	The 0.5 mg L^−1^ trans-zeatin riboside (TZR) dose with 16 h of light was the best treatment for seedling growth.	Sprouts grown from seeds (10.0 mm)	[25]
*V. floribundum*	WPM	2.0 mg L^−1^ of indolbutyric acid (IBA) for rooting.	Seedlings obtained in in vitro propagation	[25]
*V. floribundum*	mWPM (modified Woody Plant Medium)	The 5.0 mg L^−1^ 2iP and 0.1 mg L^−1^ NAA dose resulted in a significantly higher number of buds per bud.	Lateral buds	[17]
*V. vitis-idaea* ssp. *minus* (Lingonberry)	semi-solid medium BM-D	The 2.0 mg L^−1^ zeatin and 0.4 mg L^−1^ TDZ doses were the most effective for adventitious shoot regeneration.	Leaf explants	[42]
*V. corymbosum* ‘Legacy’	WPM	1.60 mg L^−1^ 2iP for sprout initiation.	The first four plant segments	[40]
*V. corymbosum* ‘Sunshine Blue’	WPM	2.0 g L^−1^ zeatin (ZEA) and 250.0 mg L^−1^ or 500.0 mg L^−1^ indolbutyric acid (IBA) for callus and adventitious shoot induction.	Callus, leaf and stem explants	[41]
*V. corymbosum* ‘Sunshine Blue’	WPM	2.0 g L^−1^ indolbutyric acid (IBA) for rooting.	Microshots	[41]
*V. corymbosum* ‘Duke’ and ‘Hortblue Petite’	WPM (bioreactor platform)	0.5 or 1.0 mg L^−1^ zeatin (ZEA) and 5.0 mg L^−1^ 2iP	Outbreaks	[8]

### 3.3. In Vitro Culture Techniques for V. floribundum Kunth

The articles that address in vitro culture techniques for *V. floribundum* Kunth (n = 3) mention that this species is coveted for its economic and agronomic potential, especially in regions such as Ecuador. Conventional propagation of this species has been complex, which has led to the search for more efficient in vitro culture methods. To achieve in vitro micropropagation, it is essential to use specific culture media such as WPM [39] and to regulate environmental factors such as light and temperature, which are crucial for seed germination and shoot growth of *V. floribundum* Kunth.

According to Cobo et al. [17], axillary shoot proliferation and adventitious shoot regeneration are important techniques for the micropropagation of *Vaccinium* species, with morphogenesis being highly dependent on the plant growth regulators and culture media used, which are genotype-specific. The combination of cytokinins such as trans-zeatin riboside (TZR) and auxins such as naphthaleneacetic acid (NAA) has been shown to be effective for the culture of germinated seedlings in vitro and for axillary shoot growth. For seedling elongation and rooting, a basal medium without hormones or with 2iP (6-(gamma,gamma-dimethylallylamino) purine) has been used, which has allowed for success in these stages of the micropropagation process.

The in vitro propagation of the species has been affected by the presence of harmful microorganisms in wild mulberry explants. These microorganisms have been particularly difficult to eliminate with simple disinfection methods, so the use of N-[1H-benzimidazole-2-yl] methyl carbamate has been proposed for the disinfection of cuttings in micropropagation studies of *V. floribundum*. In addition, light has been reported to improve germination [19].

Three successful in vitro culture techniques are presented:

#### 3.3.1. Axillary Buds

In vitro propagation through axillary bud culture has established itself as a fundamental strategy for efficient clonal multiplication of several *Vaccinium* species, including the highbush blueberry (*V. corymbosum* L.), low blueberries (*V. angustifolium* Ait.), rabbiteye blueberries (*V. ashei* Reade), wild blueberries (*V. myrtillus* L.) and other species of agronomic and conservation interest [13,33]. This technique takes advantage of the totipotency of plant cells present in axillary buds to generate a large number of plants genetically identical to the mother plant in a controlled environment [5].

The technique consists of selecting young and vigorous shoots from healthy mother plants, preferably in the juvenile stage, as it has been reported that explants from young plants present higher in vitro responsiveness [40]. Nodal segments containing one or two axillary buds are isolated, varying in length from 0.5 to 2.0 cm depending on the species and protocol [37,44].

Subsequently, a thorough disinfection process is performed to eliminate epiphytic microorganisms. This usually includes sequential immersions in ethanol solutions (70% *v*/*v*) and a sodium hypochlorite (NaOCl) solution with an optimized concentration and exposure time (3% *v*/*v* NaOCl for 15 min). Explants were washed with sterile deionized water three times for 15 min [36,37]. The addition of a few drops of Tween-20 can improve wetting and sterilization efficiency [37].

Sterilized explants are placed in nutrient culture media specific for woody plants, with the Woody Plant Medium (WPM) being one of the most used and efficient for the genus Vaccinium due to its low salt content [13,27,40]. The medium is supplemented with sucrose as a carbon source (generally between 20 and 30 g L^−1^) and a gelling agent such as agar (5–8 g L^−1^). A crucial aspect is the incorporation of plant growth regulators, mainly cytokinins, which promote the sprouting and multiplication of axillary buds. The most commonly employed cytokinins include 6-benzylaminopurine (BAP), zeatin (ZEA) and 2-isopentenyladenine (2iP) in concentrations that vary according to species and cultivar [5,13,27]. In some cases, a low concentration of auxin (such as indole-3-acetic acid (IAA) or naphthaleneacetic acid (NAA)) can be added to enhance shoot multiplication [13,17].

Once multiple shoots have formed, they can be transferred to a fresh medium with a slightly lower or even cytokinin-free concentration to promote elongation [45]. Elongated shoots (usually 1.5 to 3.0 cm in length) are separated and transferred to a rooting medium. This medium usually has a reduced salt concentration (e.g., half MS or half WPM) and is mainly supplemented with auxins, with indole-3-butyric acid (IBA) being the most effective auxin for root induction in *Vaccinium* [13,25]. The concentration of IBA varies between 0.5 and 4.0 mg L^−1^, depending on the cultivar [46]. In some protocols, the addition of activated charcoal (0.1–0.2%) to the rooting medium has been shown to improve the quality and percentage of rooting [36,37]. Rooting can also be performed ex vitro by immersing the microshoots in 500 mg L^−1^ solutions of IBA for 24 h before planting in a substrate [13,17].

Rooting can also be performed ex vitro by immersing the microshoots in 500 mg L solutions of IBA for 24 h before planting in a substrate.

Seedlings rooted in vitro are gradually transferred to ex vitro conditions in an environment of high humidity and controlled temperature (20–25 °C) [14]. Substrates such as peat alone or in mixtures with perlite or vermiculite, which provide good drainage and aeration, are used [14,17,25]. Humidity is progressively reduced over a period of several weeks to allow the plants to adapt to the ex vitro environment.

This technique has been successfully applied to a wide range of *Vaccinium* cultivars, with variations in multiplication and regeneration rates depending on the genotype, explant type, culture medium and combination of plant growth regulators used.

In cultivars ‘Farthing’ and ‘Legacy’, WPM supplemented with different concentrations of BAP (2.0 to 8.0 mg L^−1^) using both shoot tips and two-node explants (containing axillary buds) showed significant shoot production. For ‘Farthing’, concentrations of 6.0 or 8.0 mg L^−1^ BAP were effective, whereas for ‘Legacy’, 4.0 mg L^−1^ BAP was optimal [27].

In the cultivar ‘Duke’, different concentrations of zeatin (Z) and 2iP were evaluated in WPM, obtaining variable proliferation rates in solid and liquid culture systems (temporary immersion bioreactor—TIS). The cultivar ‘Hortblue Petite’ showed higher biomass production compared to ‘Duke’ [8].

In the cultivar ‘Bluejay’, axillary shoot induction was achieved on the Anderson medium supplemented with ZEA (13.68 μM) and NAA (0.27 μM). Subsequent shoot multiplication was optimized on the same medium with ZEA (9.12 μM) and NAA (0.05 μM) [13].

**Figure 2:**
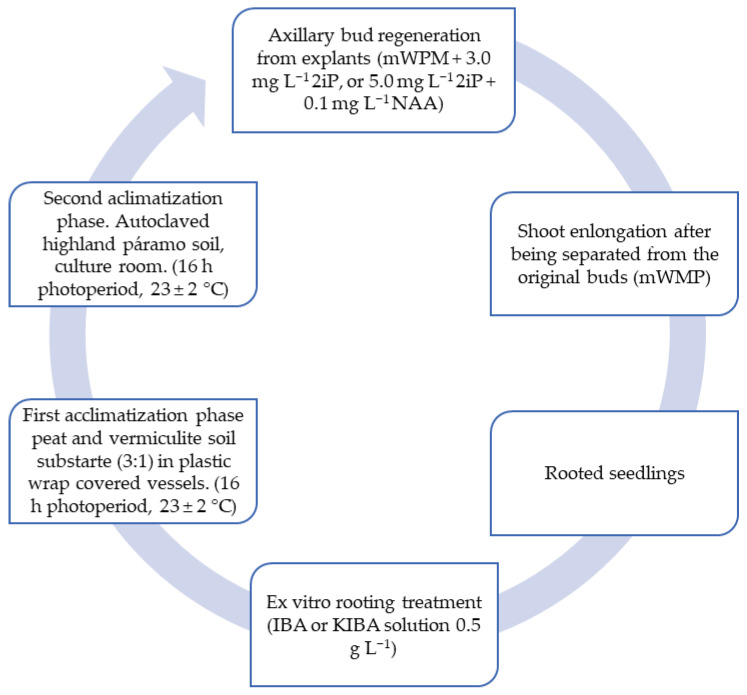
Regeneration of mortiño (*V. floribundum* Kunth) plants using axillary bud culture [17].

For mortiño, apical stem segments are used as the plant material. According to Cobo et al. [17], as shown in Figure 2, an efficient propagation methodology for *V. floribundum* via axillary bud growth is presented. A significantly higher number of shoots per bud is obtained in the modified Woody Plant culture medium (mWPM) with 2iP and NAA. In vitro rooted plants acclimatize successfully in a peat–vermiculite substrate, while unrooted plants grow efficiently after ex vitro rooting treatment by immersion in the 0.5 g L^−1^ indole-3-butyric acid (IBA) or potassium IBA (KIBA) solution.

The regeneration rate of axillary buds is 36% with a survival rate in the first acclimatization phase of 93.75%, as detailed in Table 3.

Regarding the regeneration of axillary buds and the elongation of apical stem segments, the mWPM culture medium with growth regulators 2IP and NAA at a concentration of 5.0 mg L^−1^ and 0.1 mg L^−1^, respectively, was used, maintaining a temperature of 23 ± 2 °C and a photoperiod of 16 h for 16 weeks. The regeneration rate was 36%, with an average of 9 shoots per bud and a shoot size of 18.13 mm. In the rooting phase, IBA/KIBA with a concentration of 0.5 g L^−1^ was used, with an incubation temperature of 18 °C for 24 h, achieving a survival rate of 46.67%. During the first acclimatization phase, a substrate of peat and vermiculite was used in a 3:1 ratio, with a photoperiod of 16 h, a temperature of 23 ± 2 °C and relative humidity between 30 and 90%, achieving a survival rate of 93.75% in a period of 6 to 8 months. In the second acclimatization phase, moorland soil was used under the same photoperiod and temperature conditions.

#### 3.3.2. Seed Germination

In vitro seed germination is a fundamental technique in the propagation of various *Vaccinium* species, offering significant advantages over conventional methods, especially in overcoming germination barriers and increasing seedling viability in the early stages of development [43]. The technique is based on the aseptic sowing of seeds in a defined nutrient culture medium under controlled environmental conditions [5].

Therefore, contamination by microorganisms in seeds must be prevented, and for this purpose, several protocols are presented, which commonly include immersion in ethanol, followed by a solution of sodium hypochlorite (NaOCl) for a certain time and subsequent rinses with sterile distilled water [3,39,46].

Sterilized seeds are placed in a specific culture medium for woody plants (WPM) [28,40] or Murashige and Skoog (MS) [25] supplemented with sucrose as a carbon source and agar as a gelling agent in most cases, although liquid media can also be used [46]. The pH of the medium is typically adjusted to a slightly acidic range, such as 5.0 for *V. corymbusum* [40].

The containers with the seeds are incubated in growth chambers at a specific temperature (usually between 18 and 25 °C) and photoperiod (from total darkness to 24 h of light), depending on the species and the objectives of the study [14,25,30,37,46]. Light intensity is also monitored [14,30], and the percentage of germinated seeds and the time required for germination are recorded [25].

This technique has been successfully applied to several *Vaccinium* cultivars and hybrids. In interspecific hybrids of *V. uliginosum* × (*V. corymbosum × V. angustifolium*), in vitro germination percentages of 42% were obtained for the hybrid ‘Northcountry’ (V.ul.8 × ‘NC’) and 88% for the hybrid ‘SC 5-8′ (V.ul.8 × ‘SC 5-8′) [43]. Importantly, while germination on the soil substrate for similar hybrid combinations was 80%, survival did not exceed 2%, underscoring the advantage of in vitro technologies for preserving unique genetic materials [43].

For *V. myrtillus* (wild blueberry), an in vitro grown seed germination percentage of 87.5% was reported at a temperature of 22.5 °C [47]. Seeds started to germinate within 15–20 days on the medium [47].

**Figure 3:**
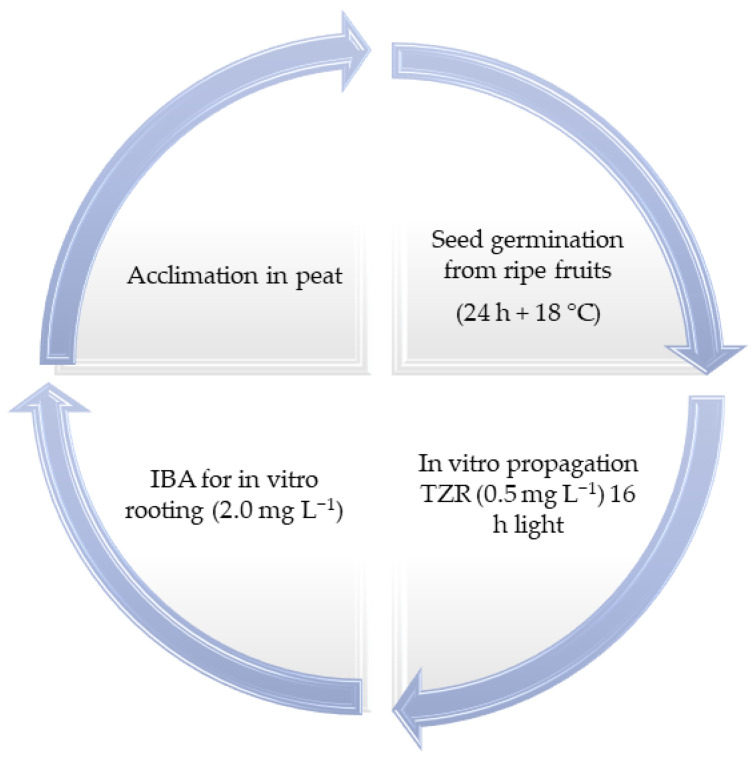
In vitro seed production of *V. floribundum* plants [25].

For mortiño, a detailed study revealed that the seeds are used as the plant material for promissory technology. The seeds of *V. floribundum* should be collected from mature fruits in the Andean highlands. The in vitro germination of *V. floribundum* seeds should be carried out under controlled light and temperature conditions (24 h light and 18 °C). The cytokinins TZR (Trans-zeatin) and ZEA (Zeatin) are efficient for the multiplication of *V. floribundum* seedlings via organogenesis. Rhizogenesis is achieved using IBA (butyric acid) at a concentration of (2 mg L^−1^). To harden the plants in vitro, they must be transplanted to peat as the soil substrate, so this technique can be used for the restoration of *V. floribundum* populations in disturbed Andean paramos in Ecuador [25]. The in vitro protocol for *V. floribundum* is synthesized in Figure 3.

Table 4 shows the results obtained from seed germination and seedling micropropagation, where WPM and MS culture media were used under a photoperiod of 24 h of light and a constant temperature of 18 °C. Seeds showed a germination percentage of 59.60%, with radicle emergence at 10 days, plumules at 30 days and true leaves between 40 and 45 days. For micropropagation, the growth regulator TZR was used at a dose of 0.5 mg L^−1^, achieving a shoot length of 10.10 mm and a multiplication rate of 6.5 shoots per explant in a period of 6 weeks under 16 h of daily light. Rooting induction was performed with IBA at a dose of 2.0 mg L^−1^, obtaining 100% rooting and a root length of 10.20 mm. During in vitro acclimatization, peat was used as the substrate, and an increase in plant length of 3.28 mm was observed without the need for fertilization, with 100% survival.

In vitro seed germination allows us to overcome natural barriers to germination, such as dormancy, and provides a protected environment against pathogens and unfavorable environmental conditions, leading to a higher seedling survival rate compared to substrate germination [43]. In addition, it facilitates obtaining a large number of seedlings in a small space and under controlled conditions, which is crucial for research, germplasm conservation and mass propagation [5].

#### 3.3.3. Induced Callogenesis

The induction of callus, a mass of undifferentiated parenchyma cells, has become a fundamental step in several in vitro culture techniques applied to the genus *Vaccinium*. The ability to generate and manipulate callus allows for the implementation of protocols for genetic transformation, the induction of polyploidy and the regeneration of plants with improved characteristics.

The process consists of using various parts of the plant as explants, with field-grown mature leaves being the most common due to their robustness and high regeneration capacity [10]. Leaf disks, stem segments and even somatic embryos have also been used [7,45]. Thorough sterilization of the explant is crucial to eliminate microbial contamination without compromising tissue viability [10]. An effective protocol includes sequential immersions in 75% alcohol and an 8% sodium hypochlorite (NaOCl) solution, optimizing the exposure time for each explant type and cultivar [10,36].

Sterilized explants are placed in a nutrient culture medium, with the Woody Plant Medium (WPM) being one of the most commonly used since its composition is suitable for woody species [10,40,45]. Callus induction is achieved via the incorporation of plant growth regulators (PGRs), mainly auxins, often in combination with cytokinins.

2,4-dichlorophenoxyacetic acid (2,4-D) and indole-3-butyric acid (IBA) are commonly employed auxins for callus induction in *Vaccinium* [6,9,10,45]. Concentrations vary according to the cultivar and explant. Cytokinins such as forchlorfenuron (CPPU) and 2-isopentenyladenine (2iP) have also been shown to be effective for the induction and growth of callus on blueberry leaves [45]. In some cases, zeatin (ZEA) is used in combination with auxins for callus induction and subsequent shoot regeneration [10].

Explants are grown under controlled conditions of temperature (usually 24–25 °C) and photoperiod (often initial darkness followed by a 16/8 h light/dark cycle) [10,45]. The formed corns are periodically subcultured on a fresh medium with the same or different hormone composition to promote their growth and prevent the browning or loss of regenerative capacity.

Callus induction in *Vaccinium* has provided a solid basis for *Agrobacterium tumefaciens*-mediated transformation that has allowed for the introduction of genes of interest for improving traits such as herbicide and cold stress resistance [2,10]. The identification of cultivars with high callus formation and regeneration ability, such as *V. reticulatum* ‘Red Button’, is crucial to advance functional genetics studies and breeding programs [10]. The application of agents such as colchicine to plant tissues, often at the callus stage or during indirect organogenesis, is used to induce chromosomal duplication and generate polyploid plants with potentially improved characteristics [28,30]. In addition, it allows for the large-scale clonal multiplication and recovery of genetically modified or polyploid plants [3,33,45]. Callus culture can also be used for the production and study of secondary metabolites of pharmaceutical interest [8].

**Figure 4:**
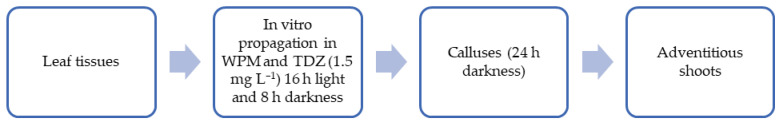
Callus induction from *V. floribundum* leaf tissues [24].

According to Meneses et al. [24], callogenesis is an efficient in vitro regeneration alternative for *V. floribundum* Kunth. Where callus formation was induced from the leaf tissue of in vitro mortiño plants. The explants should be grown in the WPM culture medium supplemented with Thidiazuron cytokinins (TDZ) at a concentration of 1.5 mg L^−1^. The plant material should be incubated at 16 h of light and 8 h of darkness to obtain 90% callus formation. The protocol used to induce callus formation is summarized in Figure 4. Callus can be produced from a variety of explants, including meristematic and non-meristematic explants. Callus is the starting material for embryo generation or de novo organ development and thus acts as a basis for the plant biotechnology approach that allows for the rapid multiplication of plants under sterile conditions [48,49].

This technique generates the oxidation of leaf tissue, which is why Table 5 shows the number of oxidized leaves together with the percentage of callus formation and the time where adventitious shoots are obtained.

Leaf tissue was provided with the WPM culture medium combined with TDZ; it was observed that the oxidation of leaf tissue affected an average of 1.16 leaves. In addition, 90% callus formation was achieved, and adventitious shoot emergence occurred within 10 days. The plants were exposed to a photoperiod of 16 h of light daily, which contributed to the observed results. These findings highlight the efficacy of the WPM+TDZ growing medium in promoting callus formation and adventitious shoot development under controlled light conditions.

## 4. Conclusions

The in vitro regeneration of *Vaccinium floribundum* Kunth can be effectively carried out via axillary budding, callogenesis and seed germination using the modified woody plant medium supplemented with 5.0 mg L^−1^ of 2-isopentenyladenine and 0.1 mg L^−1^ of 1-naphthaleneacetic acid together. This combination not only enhances shoot proliferation but also promotes the acclimation of rooted seedlings. These findings are consistent with regeneration trends observed in other *Vaccinium* species, highlighting the importance of customized combinations of growth regulators and culture media for optimal results.

However, it is crucial to highlight the limited number of studies currently available on the in vitro propagation of *Vaccinium floribundum*. Addressing this gap is essential from a biodiversity conservation point of view, especially as this species is still mainly exploited through wild collection. The development of robust micropropagation protocols could make an important contribution to alleviating the pressure on natural populations and supporting sustainable commercial utilization. In addition, available in vitro methodologies applied to other *Vaccinium* species may provide useful information to improve *Vaccinium floribundum*-specific propagation techniques. Therefore, further research is needed to search for new growth regulators and to optimize acclimatization methods.

## Figures and Tables

**Table 1:** *Vaccinium* species studied.

Species (*Vaccinium* spp.)	Main Geographic Location	Climatic Aspects	References
*V. corymbosum* L. (High Blueberry)	Poland, Romania, Chile and USA.	Adaptable to various conditions and needs winter cold accumulation in some varieties.	[29]
*V. ashei* Reade (Bilberry Rabbiteye)	South Korea and Brazil	It grows in subtropical climates with mild winters.	[38]
*V. myrtillus* L. (European Blueberry)	Austria and Turkey (Black Sea region)	Grows in natural populations.	[33,34]
*V. floribundum* Kunth (Mortiño)	Andean highlands of South America (Ecuador)	Grows wild in moorland and high-altitude conditions.	[25]
*V. arboreum* (Sparkleberry)	USA (Texas)	Grows in municipal parks.	[35]
*V. uliginosum* L. (Swamp Blueberry)	Turkey	It grows in natural populations at high altitudes (2760 m).	[36]
*V. arctostaphylos* L.	Iran (Hyrcanian forests), Turkey (Black Sea region) and Bulgaria.	Grows as a perennial shrub in specific floras.	[37]

**Table 3:** Results and characteristics of in vitro *V. floribundum* Kunth cultivation using axillary buds.

Plant Material	Axillary Bud Regeneration		Elongation		Reference
**Apical stem segments**	Culture medium	mWPM	Culture medium	mWPM	[17]
	Growth regulators	2iP + NAA	Time	16 weeks	
	Dosage	5.0 mg L^−1^ + 0.1 mg L^−1^			
	Temperature	23 ± 2 °C	**In Vitro and ex vitro rooting**		
	Photoperiod	16 h	IBA/KIBA	0.5 g L^−1^	
	Time	16 weeks	Incubation temperature	18 °C	
	Regeneration rate	36%	Time	24 h	
	Average number of shoots per bud	9			
	Shoot size (mm)	18.13			
	Growth rate	0.76			
	**First acclimation phase**		**Second acclimation phase**		
	Substrate	Peat and vermiculite	Substrate	Páramo soil	
	Ratio	3:1	Photoperiod	16 h	
	Photoperiod	16 h	Temperature	23 ± 2 °C	
	Temperature	23 ± 2 °C	Survival rate	46.67%	
	Relative humidity	30 and 90%			
	Time	6–8 months			
	Survival rate	93.75%			

**Table 4:** Results and characteristics of *V. floribundum* seed germination.

Plant Material	Seed Germination		Micropropagation of Seedlings		Reference
Seeds	Culture medium	WPM and MS	Growth regulator	TZR	[25]
	Photoperiod	24 h light	Concentration	0.5 mg L^−1^	
	Temperature	18 °C	Shoot length	10.10 mm	
	Germination	59.60%	Light	16 h	
	Germination time	10 days= radicle 30 days = plumules 40–45 days= true leaves	Multiplication rate	6.5 shoots per explant	
			Time	6 weeks	
	**Rooting induction**		**Acclimatization in vitro**		
	Growth regulators	IBA	Substrate	Peat	
	Concentration	2.0 mg L^−1^	Increase in plant length	3.28 mm	
	Rooting	100%	Fertilization	It is not necessary	
	Root length	10.20 mm	Plant survival	100%	

**Table 5:** Results and characteristics of *V. floribundum* callus induction.

Plant Material	WPM + 1.5 mg L^−1^ TDZ		Reference
Leaf tissue	Oxidation of leaf tissue (number of leaves)	1.16	[24]
	Callus formation %	90	
	Adventitious shoots	10 days	
	Photoperiod	16 h light	

## Abbreviations

The following abbreviations are used in this manuscript:

2,4-D	2,4-dichlorophenoxyacetic
2iP	2-isopentenyladenine
AC	Activated carbon
AN	Anderson medium
BAP	6-benzylaminopurine
C2D	Chee and Pool medium
CPPU	Forchlorfenuron
GA3	Gibberellic acid
IAA	Indole-3-acetic acid
IBA	Indole-3-butyric acid
KIBA	Potassium indole-3-butyric acid
MS	Murashige and Skoog
mWPM	Modified Woody Plant Medium
NAA	Naphthaleneacetic acid
NaOCl	Sodium hypochlorite
PDMCs	Plant Derived Medicinal Compounds
PGRs	Plant growth regulators
PTC	Plant tissue culture
RITA	Recipient for Automated Immersion Technique
TDZ	Thidiazuron cytokinins
TISs	Temporary immersion systems
TZR	Trans-zeatin
WPM	Woody Plant Medium
ZEA	Zeatin
